# Tuberculosis diagnostic and treatment delays among patients in Uganda

**DOI:** 10.1002/hsr2.1700

**Published:** 2023-11-15

**Authors:** Emmanuel I. Obeagu

**Affiliations:** ^1^ Department of Medical Laboratory Science Kampala International University Kampala Uganda

**Keywords:** diagnosis, patients, treatment delays, tuberculosis

## Abstract

**Background:**

*Mycobacterium tuberculosis*, a bacterium that relies on its human host to achieve airborne transmission and existence, is the primary cause of tuberculosis (TB), a disease that is vital to public health.

**Aim:**

To update the society on tuberculosis diagnostic and treatment delays among patients in Uganda.

**Materials and Methods:**

The review paper utilized different search engines, such as Pubmed Central, Scopus, Web of Science, Google Scholar, and so forth, to conduct this review paper.

**Results:**

Delays in diagnosis could cause diseases to spread throughout the community, progress more quickly, and increase mortality. With many populations experiencing TB diagnostic delay and less than a third of the population experiencing TB treatment delay, the rates of tuberculosis diagnosis and treatment delays are high.

**Conclusion:**

The delay in diagnosing and treating tuberculosis in men is positively correlated with knowledge of the disease's symptoms and the regular use of a handkerchief or both hands to cover the mouth and nose while coughing or sneezing.

## 1 INTRODUCTION

*Mycobacterium tuberculosis*, a bacterium that relies on its human host to achieve airborne transmission and existence, is the essential cause of tuberculosis (TB), a disease that affects public health.^1^, ^2^, ^3^, ^4^, ^5^, ^6^ Since more than 50 years ago, efforts have been made to eradicate tuberculosis (TB), one of the oldest human diseases.^7^ After HIV, it is the second most common cause of infectious disease death worldwide, but it is still curable.^8^, ^9^, ^10^

One of the regions in Sub‐Saharan Africa with the highest tuberculosis rates is the Lake Victoria region.^11^ 91.2 million people worldwide contract tuberculosis annually, according to the World Health Organization (WHO),^7^ with 95% of tuberculosis patients living in low‐income nations.^12^ Tuberculosis is a major infectious cause of morbidity and mortality worldwide, and in 2017 it was predicted that there would have been about 10 million new cases and 1 point six million deaths from the disease.^12^ Uganda, one of the 22 countries with the highest tuberculosis burden, has a 67% treatment success rate, and 50% of TB patients are also HIV coinfected.^13^ Since the disease burden is disproportionately concentrated in low‐ and middle‐income countries, these nations account for about 95% of all TB‐related deaths.^14^ Despite the fact that early diagnosis and treatment could have prevented the majority of these deaths, there were still close to 40% of undiagnosed TB cases worldwide by 2018.^15^

The number of untreated cases of tuberculosis is a significant problem, and it is getting worse all over the world.^16^ This could be due to a number of factors, including the time it takes between the onset of the disease and the diagnosis and the beginning of treatment.^17^ Delay in diagnosis may result in disease spread throughout the community, an increase in mortality, and the progression of the illness.^18^, ^19^, ^20^, ^21^

Significant strides in reducing tuberculosis morbidity and mortality have been made over the past 10 years.^16^ To lessen exposure to and contact with contagious individuals, limit outpatient visits and encourage patients to quickly seek care if they experience symptoms resembling tuberculosis.^22^

A number of problems, such as expensive medical care, limited access to healthcare, and social stigma, have contributed to the delay in diagnosis and insufficient uptake of tuberculosis treatments.^23^ Risk factors that contribute to the spread of the disease and social and economic instability include overcrowding and unhygienic living conditions.^24^ Earlier study looked at patient and community characteristics (health‐seeking behavior) that affect delays in diagnosis and treatment initiation rather than the delay that occurs once a symptomatic patient contacts the healthcare system.^25^

Delays in tuberculosis diagnosis and treatment are related to social, demographic, and knowledge factors. Living in an urban slum increases your risk of developing tuberculosis compared to the general population, which has been shown to influence how often you seek out medical care. While examining the knowledge of tuberculosis and its associated socio‐demographic variables, researchers discovered a low understanding of TB causation, symptoms, transmission, prevention, and free treatment. On tuberculosis treatment and prevention, there was a decent amount of knowledge. Poor knowledge of tuberculosis was caused by a variety of independent factors, including lack of formal education, unemployment, and never having an HIV test.^26^

A study in Mumbai that examined the factors that contributed to simple pulmonary TB patients' delayed diagnosis found that significantly more female patients experienced this.^13^ A cross‐sectional study conducted at the Yangon Regional Tuberculosis Center in Myanmar discovered that incomplete middle school education, temporary employment, co‐existing diabetes mellitus, and a lack of knowledge were some of the predictors of a protracted diagnosis delay. Age between 31 and 50, a history of MDR‐TB patients older than 50, living more than 20 km from a regional tuberculosis center, and ignorance were all independently predictive factors for protracted treatment delays.^12^ An analysis of newly diagnosed pulmonary TB cases registered in 2010 at the Ahvaz Health Center was done in a retrospective fashion to determine the overall treatment delay and its contributing factors. Smoking, being a woman, and taking immunosuppressive drugs were all significantly correlated with delayed time.^25^

A cross‐sectional study of new patients receiving care from the National Tuberculosis Control Programme was conducted in 70 randomly selected Vietnamese districts. revealed that patients with significant overall delays accounted for 49% of the total number of delay‐weeks. The first visit to the private sector, being a woman, being in your middle years, living in a remote area, living in the northern or central part of the country, and being of middle age were all independent risk factors for a lengthy total delay. Long patient wait times were caused by female sex, ethnic minority status, and living more than 5 km from a hospital or in a remote location. A first visit to a public health facility, a tuberculosis hospital, or a private facility was included in this.^14^ Other factors included residing in a city, being in the middle, and making a first visit. A second cross‐sectional investigation was conducted in Uzbekistan on hospitalized patients with recently diagnosed tuberculosis. Among the 538 people who were recruited, the median interval between the beginning of symptoms and the beginning of anti‐TB medication was 50 days. Analysis of the factors influencing health‐seeking behavior and prompt treatment demonstrated the existence of the patient factor. Self‐medication was the first health‐seeking behavior for 231 patients and was found to be a significant predictor of delay, along with coughing, weight loss, and visiting both primary and private healthcare facilities.^27^

Sendagire and associates.^24^ carried out a study in Kampala, Uganda, at public primary healthcare facilities to determine the prevalence of diagnostic delay among patients with pulmonary tuberculosis and to assess contributing factors. According to the study, the average wait time for all diagnostic procedures was 8 weeks. If patients knew tuberculosis was curable, they were less likely to encounter a lengthy total delay and patient. Women, people who have lived at their current address for more than 5 years, and people who have previously had an HIV test all had longer wait times for medical care. 91% of patients visited one or more healthcare providers on average four times before receiving a diagnosis. In the majority of patients, systemic symptoms were present by the time tuberculosis was diagnosed. Failure to recognize symptoms was the primary reason for a delayed diagnosis, according to a second study conducted among Ugandan women. Due to low levels of tuberculosis awareness in the local population, this may have occurred. The study also showed that there is tuberculosis stigma in Uganda, primarily because of links to HIV. Numerous participants believed that tuberculosis only coexisted with HIV and that tuberculosis caused HIV tests, even in those who were HIV‐infected, to come back negative.^28^

## 2 TUBERCULOSIS PATHOGENESIS AND PROGRESSION

Aerosol droplets containing *M. tuberculosis* that are released when active tuberculosis patients cough, sneeze, or talk are how tuberculosis is spread.^29^ The tuberculosis bacteria enter the new host's airways and pass through the respiratory system to the lung. The host's innate immune system now kicks in to stop the infection, and alveolar macrophages then internalize the tubercle bacilli. Bacilli multiply inside their intracellular environment when macrophages fail to inhibit or kill them, are released, are phagocytosed by additional alveolar macrophages, and the cycle repeats.^29^ Following the recruitment of lymphocytes to the infection site, a cell‐mediated immune response is launched in an effort to contain the bacteria and stop their further growth.^30^ The host is still asymptomatic at this point, and the tuberculosis bacteria may be completely eradicated or enter latency within the granuloma.^31^ However, when immunity is compromised, the illness quickly progresses to active tuberculosis with clinical symptoms.^31^

### 2.1 Pulmonary tuberculosis' pathophysiology

The bacilli enter the lung after *M. tuberculosis* transmission to the new host and are ingested by macrophages. To wall off the infected macrophages, additional immune cells are enlisted, which causes the granuloma—the tuberculosis granuloma—to form. At this point, the infection is kept at bay in healthy individuals who are still latently infected, but it is still susceptible to the risk of reactivation. When foamy macrophages necrotize, their lipid content is released, resulting in caseation. Caseum is a decay that appears at the granuloma's core and compromises the structure's rigid integrity. Bacilli start to leak out of macrophages and into the caseum layer as the granuloma grows. The reactivation causes *M. tuberculosis* to multiply and the bacterial load to soar, which causes the granuloma to rupture and spread the bacteria to the airways. After restarting the cycle and infecting additional people, the bacilli expel themselves as infectious aerosol droplets.

The granuloma, an amorphous collection of macrophages and other immune cells meant to stop the spread of the bacteria, is the key characteristic of pulmonary tuberculosis.^31^ In immunocompetent people, the granuloma restrains the bacilli and prevents the development of the active disease even though it is unable to eradicate the pathogen.^30^ The bacteria manage to avoid dying, however, by preventing phagolysosome fusion and manipulating the host's immune response. By tricking the immune system and surviving in a nonreplicating or slowly replicating state, *M. tuberculosis* can survive for decades in this process‐created hospitable niche.^30^ The patient in this instance is latently infected but still noninfectious and asymptomatic. Targeting this persistent pathogen inside the granuloma is notably one of the difficulties facing the current tuberculosis therapy.

Macrophages change into foamy macrophages and other morphotypes as the granuloma matures.^32^ The host immune cells' necrotic lysis may cause the granuloma's center to necrotize, resulting in the formation of a caseum (caseous granuloma).^33^ The caseum, or accumulating soft necrotic debris, is what gives the granuloma's core its name because it resembles cheese. Around the necrotic foci of the granuloma, foamy macrophages, which are distinguished by accumulated lipid droplets, are distributed.^34^ Importantly, it was discovered that the dysregulation of host lipid metabolism caused by *M. tuberculosis*, which occurs when the balance between the sequestration and efflux of lipid particles from the serum is upset, is a key factor in the development of the illness.^32^ The disruption of lipid metabolism encourages the growth of foam cells, which help bacteria survive and ultimately lead to the buildup of caseum in the granuloma.^32^ Additionally, it has been suggested that mycolic acids (MAs), which are the primary lipid components of the *M. tuberculosis* robust cell wall and are necessary for mycobacterial growth and survival, aid in the differentiation of macrophages into foam cells.^35^, ^36^ The resulting caseous lesions act as reservoirs by containing and protecting the tubercle bacilli, which keep the bacteria in their dormant state.^37^ The patient develops active tuberculosis, which results in the transmission of the infectious bacilli into a new host, but in the late stages of the disease, the caseous core softens and cavitation occurs, reviving the bacteria.^32^, ^37^ The ability of the host's immune system to prevent bacterial replication is crucial to this potentially fatal transformation.^30^

Even though HIV co‐infection is the primary factor in tuberculosis reactivation, other factors can also make the infection active. Malnutrition, immunosuppressive drugs, chemotherapy, uncontrolled diabetes mellitus, sepsis, drug or alcohol addiction, chronic renal failure, smoking, and cancer are a few of these triggers.^30^ The dormant bacilli that were initially contained in the granuloma will reactivate and replicate when the host has compromised immunity, which will be accompanied by the granuloma liquifying and cavitating.^34^ As a result, the granuloma's structure deteriorates, and the infectious bacteria are released, causing cavitary lesions to form and symbolizing the lung damage seen in tuberculosis patients.^30^, ^38^ Additionally, the caseous material acts as a rich source of nutrients that encourage the pathogen to grow into a burdensome size.^38^ Finally, the bacilli disperse throughout the lung and reach the blood capillaries, opening the door for dissemination to other organs in addition to transmission to other people.^38^ At this point, the condition (active tuberculosis) becomes very contagious and manifests symptoms. The progression of granulomas is believed to be correlated with tuberculosis reactivation because lung histology during the active disease shows the coexistence of granulomas at various stages of development.^38^ While it has been determined that there are three main types of granulomas, namely solid, necrotic, and caseous granulomas, they actually form a continuum and shouldn't be thought of as separate things. Histologically, solid granulomaform correlates with both the pathology and the containment of *M. tuberculosis* because it occurs early in the course of the disease and involves tissue damage. It is typically surrounded by a fibrotic wall, lacks central necrosis, and contains a variety of immune cells, particularly T lymphocytes, which are the main agents in containing the infection.^38^ Granulomas in solid form are common in latent tuberculosis infection because the *M. tuberculosis* burden is low in these tumors. As the illness worsens, the center of the solid granuloma begins to necrotize (necrotic granuloma), opening the door for the reactivation of the dormant bacteria later on in the process when the necrotic center grows larger and liquifies. Granulomas from latent to active tuberculosis have previously been discussed in‐depth.^38^, ^39^, ^40^, ^41^

## 3 DELAYS IN TUBERCULOSIS DIAGNOSIS AND TREATMENT

PTB diagnoses are frequently delayed in many Sub‐Saharan African countries. Early detection of active tuberculosis is crucial for reducing population morbidity and mortality as well as nosocomial transmission in healthcare facilities. A cross‐sectional study at the Yangon Regional Tuberculosis Center in Myanmar found that the median diagnosis delay was 9 days and that 58.6% of the patients experienced a significant diagnosis delay. 51% of patients experienced lengthy treatment delays, with the median treatment delay being 13 days.^12^ Another study that looked at the health‐seeking behaviors of TB patients and people with chronic cough discovered that 75.4% of them had already sought treatment for the condition, with 59.6% of them going to a public health center and the rest to a private one. For a persistent cough, up to 13.5% of the patients visited a pharmacy. If a patient's first point of contact was a private medical facility, they had a higher chance of having a positive GeneXpert test.^42^

The two factors that led to hospitalization the most frequently among patients who were HIV‐uninfected were low Karnofsky scores and the need for diagnostic testing.^43^ Due to their low Karnofsky scores, other illnesses, and diagnostic testing, patients with HIV were frequently admitted to hospitals. Patient delay was the primary factor in the majority of delays, which in 71.6% of respondents were longer than 3 weeks. Though being a woman was found to be protective against delay, being unemployed and having little knowledge of TB were also factors associated with delay. The main, most frequent, and biggest cause of the overall delay was patient delay.^44^

## 4 CONCLUSION

Less than one‐third of the tuberculosis patients who visit hospital for diagnosis and treatment experience treatment delays, and many also experience diagnostic delays for tuberculosis. The delay in diagnosing and treating tuberculosis in men is positively related to knowledge of the disease's symptoms and regular use of a handkerchief or both hands to cover the mouth and nose while coughing or sneezing.

## AUTHOR CONTRIBUTIONS

**Emmanuel I. Obeagu**: Conceptualization; methodology; resources; supervision; validation; writing—original draft; writing—review & editing.

## CONFLICT OF INTEREST STATEMENT

The author declares no conflict of interest.

## ETHICS STATEMENT

There are no human participants in this article, and informed consent is not applicable.

## TRANSPARENCY STATEMENT

The lead author Emmanuel Ifeanyi Obeagu affirms that this manuscript is an honest, accurate, and transparent account of the study being reported; that no important aspects of the study have been omitted; and that any discrepancies from the study as planned (and, if relevant, registered) have been explained.

## Data Availability

No data was used for the research described in the article.
